# Reversible Choreoathetosis in a Patient with End-stage Renal Disease from Administration of Ceftriaxone

**DOI:** 10.7759/cureus.5764

**Published:** 2019-09-25

**Authors:** Mei Ling Tan, War War Win Tun

**Affiliations:** 1 General Medicine, Khoo Teck Puat Hospital, Singapore, SGP

**Keywords:** chorea, choreoathetosis, ceftriaxone, end stage renal disease, renal disease

## Abstract

A wide spectrum of neurological manifestations may be induced in patients with impaired renal function when receiving beta-lactam antibiotics due to the altered pharmacokinetics. Beta-lactam antibiotics is commonly chosen for treatment in patients with end-stage renal disease due to its good penetration into the cerebrospinal fluid and long half-life.

Here, we present a case of a 73-year-old Malay lady with end-stage renal disease who was admitted for treatment of gastroenteritis. She presented with acute onset of diarrhoea and vomiting for two days. She was febrile during admission and was prescribed intravenous ceftriaxone 2 grams daily for coverage of bacterial gastroenteritis. Among the investigations done, white cell count were raised together with the C-reactive protein. Stool and blood cultures were also sent for further investigations. Over a three-day period, her general condition improved and she was discharged home. The onset of clinical manifestation of choreoathetosis was noticed by her caregiver on the same day of discharge. She was brought back to the emergency department and was readmitted for further workup of the new presenting complain of abnormal movement and disorientation. Haemodialysis was arranged and immediately commenced during her admission. The renal nurses reported that her neurological symptoms were noticeably improved after completion of the initial dialysis without any treatment.

Ceftriaxone including other beta lactam antibiotics penetrates the blood-brain barrier and induces glutamate in excess in the striatum and cerebral cortex, resulting in neurological hyper excitability disorders despite appropriate renal adjusted dosage for end-stage renal disease patients on haemodialysis.

## Introduction

Patients with end-stage renal disease (ESRD) are frequently predisposed to infection due to their immune abnormalities and antibiotics agents are often prescribed [1,2]. The pharmacokinetics of many antibiotics are different in ESRD as the kidney is the major elimination pathway for many drugs and metabolites [3,4]. The third-generation cephalosporin, ceftriaxone is commonly used due to its wide coverage of gram-positive and gram-negative organism plus the long half-life making it an ideal drug of choice for convenience. Furthermore, ceftriaxone has excellent penetration into cerebrospinal fluid hence unexpected neurological side effects may be amplified [5]. Here, we present a case of a patient of advanced age with ESRD undergoing renal replacement therapy that was complicated by choreoathetosis after ceftriaxone administration for acute gastroenteritis [6].

## Case presentation

**Figure 1:**
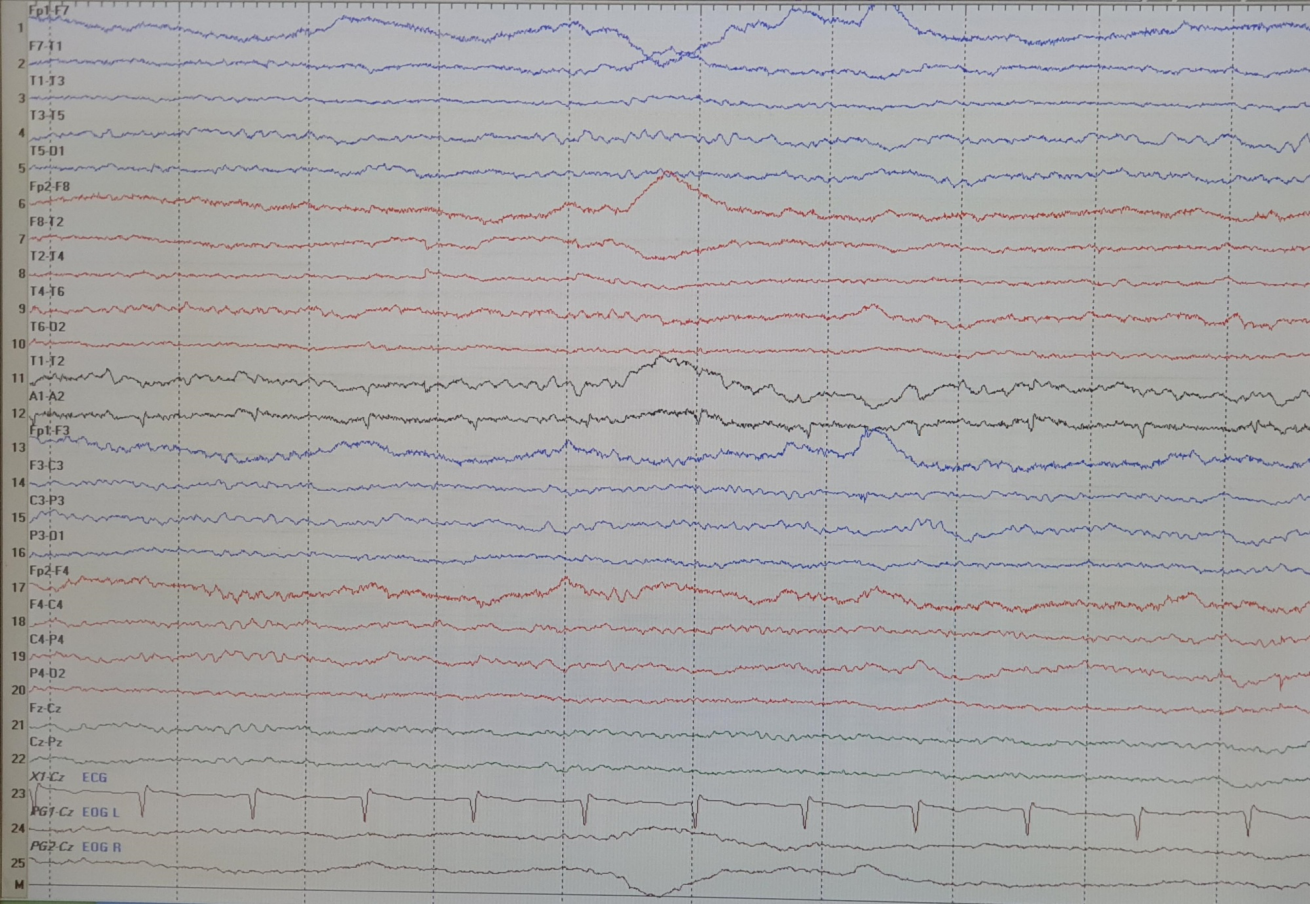
Electroencephalogram of the patient described in the case when symptoms were partially resolved after a single session of haemodialysis.

A 73-year-old Malay female had a background history of essential hypertension and type 2 diabetes mellitus, which was complicated by ESRD for the past seven years. This eventually required her to be on long-term renal replacement therapy. Initially she was initiated on peritoneal dialysis but was discontinued due to complication of peritoneal pleural leak. Subsequently, she was started on haemodialysis of three sessions per week for the past two years. She was generally well until three days prior to admission. She had high-grade fever with diarrhoea and vomiting. Clinically, she was febrile (temperature 38.1 degree Celsius) with preserved haemodynamic stability. Examination of the abdomen and other systemic review was unremarkable. On admission investigations, full blood count revealed raised total white cell count was 12.38 x 10^9^/L with neutrophils 93.3%. Haemoglobin 11.4 g/dL with normocytic normochromic picture and platelet count was 169 x 10^3^ cells/uL. Blood urea was 11.7 mmol/L and creatinine was 438 umol/L with evidence of metabolic acidosis where bicarbonate was 21 mmol/L and anion gap was 24.2. Blood glucose was acceptable at 8.3 mmol/L. Inflammatory markers were raised where C-reactive protein was 141.0 mg/L. The renal function test was comparable with the previous ones done during her outpatient follow-up visits. Two samples of blood cultures were sent for identification of causative organism together with stool culture and sensitivity. Empiric coverage for bacterial gastroenteritis was prescribed with intravenous ceftriaxone 2 g/day. Three days later, her fever lysed and diarrhoea improved with no further episodes of vomiting. All cultures were reported as no bacterial growth. Antibiotics was stopped after three days of intravenous administration. She was well upon discharge home. However, on the next morning, she developed involuntary movements involving both upper limbs and lower limbs where she was brought back to the emergency department. Her caregiver reported the onset of movements started around 15 hours after the last dose of ceftriaxone was administered. She was also disorientated and her speech could not be understood, only incomprehensible sounds were heard. She was not febrile and there were no other systemic symptoms. Clinically orofacial dyskinesia and choreiform movements were observed on all four limbs - that was worse on upper limbs. Her vital signs were stable where her blood pressure was 175/90 mmHg, heart rate was 79 beats/min and respiratory rate was 16 breaths/min. Blood chemistry was repeated including full blood count, renal function test, liver function test and bone metabolism were all unremarkably changed from baseline. A head computed tomography (CT) scan and magnetic resonance imaging (MRI) were performed but there were no abnormalities to explain these neurologic findings. The MRI showed an old cerebral infarction in the left thalamus but no acute ischemic lesions. She was diagnosed as choreoathetosis by the on-call neurologist. Considering the acute nature of her presentation, metabolic cause such as hyperglycaemia, hypercalcaemia and hyperthyroidism of acute chorea was excluded. An electroencephalogram (EEG) reported intermittent generalised background slowing indicative of mild diffuse encephalopathy with no epileptiform activity seen (Figure 1). Haemodialysis (HD) was initiated on day 2 of hospitalization and continued on alternate days. Her neurological symptoms completely disappeared without leaving any sequences after the second session of haemodialysis. She was fully orientated and her neurological function was back to her baseline.

## Discussion

In this case report, we describe an ESRD patient who presented with choreoathetosis after the administration of ceftriaxone. The diagnosis of choreoathetosis was neurologically confirmed. The relationship between the start of ceftriaxone therapy and the appearance of choreoathetosis as well as the withdrawal of ceftriaxone therapy and the disappearance of the symptom strongly indicated that ceftriaxone is a causative agent. Other causes of chorea have been excluded and no other explanation could be made for this patient.

Ceftriaxone is widely used as a beta lactam antibiotic coverage due to its broad-spectrum properties of both gram-positive and gram-negative bacteria. It has a wide distribution throughout the body including gall bladder, lungs, bone, bile, cerebral spinal fluid (CSF) at higher concentrations achieved when meninges are inflamed. The half-life elimination for adults with renal failure is prolonged to 12-16 hours and this drug is poorly dialysed including patients on intermittent haemolysis, peritoneal dialysis or continuous replacement therapy [7,8].

Common adverse effects due to ceftriaxone include local induration at injection site, generalised skin rash, gastrointestinal disorders and haematological disorder. Other more uncommon presentation including the nervous system is rarely reported. Drug-induced movement disorder with chorea is usually related to psychotic patients treated with neuroleptics and parkinsonian patients in long-term treatment with levodopa. Ceftriaxone is scarcely included due to the adverse effect [9]. Several case reports of ceftriaxone-induced choreoathetosis in ESRD patients by Sato et al. were identified [10]. Other spectrum of neurological presentation includes reversible encephalopathy after withdrawal of ceftriaxone has been reported [11,12]. Several cases of recurrent non-convulsive status epilepticus after re-exposure of ceftriaxone were also documented. These case reports support the evidence of neurotoxicity associated with ceftriaxone usage [13,14].

It is postulated that the neural mechanisms causing the drug-induced choreoathetosis could be due to the impaired gamma aminobutyric acid (GABA) modulation causing cytokine release regulated by bacterial endotoxin. Subsequently, a decrease in GABA-mediated inhibition and cephalosporin-mediated release of cytokines. Alternatively, cephalosporins may decrease GABA release from nerve terminals, increase excitatory amino acid release, and exert a competitive antagonism with GABA. Other interesting cases have been reported in rat model of Parkinson disease where excess glutamate, a neurotransmitter expression is associated with nigrostriatal neuronal damage [15,16].

Pre-existing neurological abnormalities have been indicated as a risk factor for β-lactams encephalopathy. The patient had a history of cerebrovascular disease which probably accounted for the increased risk of drug-induced encephalopathy. In most published cases of cephalosporin-induced encephalopathy, renal impairment was present. This was also the case in our patient, who had underlying ESRD and required regular haemodialysis.

Excessive dosage of cephalosporin has also been shown to be an important determinant of cephalosporin neurotoxicity [5]. Given that no dose-adjustment is required for ceftriaxone in the presence of renal failure with the dose used (2 g IV daily), excessive dosage did not seem to play a role in this case [7].

## Conclusions

In conclusion, ceftriaxone is a useful third generation cephalosporin for treating bacterial infection in ESRD, bearing in mind that it is poorly dialysed and no dosage adjustment is recommended including patients on intermittent haemodialysis with the possibility that ceftriaxone induced choreoathetosis occurring. However, several predisposing factors are to be taken into account including high dose and advanced age, as this may have serious sequalae if diagnosis is delayed.
